# The Hospital. Nursing Section

**Published:** 1902-05-10

**Authors:** 


### The Hospital.

### Nursing Section.

Contributions for this Section of "The Hospital" should be addressed to the Editor, "The Hospital" Nursing Section, 28 & 29 Southampton Street, Strand, London, W.C.

No. 815.—Vol. XXXII. *SATURDAY, MAY* 10, 1902.

### Notes on News from the Nursing World.

### THE QUEEN AND THE NURSING BOARD.

The Queen is taking a keen personal interest in the Military Nursing Service which bears her name. Her Majesty was present at the meeting of the Nursing Board last week. The proceedings at these meetings are necessarily private, but they are far from being of a merely formal character, and members of the service, as well as nurses who propose to apply for admission, will be glad to feel that in a very real sense the Queen is at the head of it.

### WAITING ON THE KING'S GUESTS.

In addition to the letters which we published last week, we have received a number of further communications from nurses in London and in various parts of the country, offering to assist in waiting upon the London poor who are to be entertained by the King shortly after the Coronation. We observe that, according to the daily press, several ladies and gentlemen have made a similar offer. Most of our correspondents want to know to whom they should apply. The arrangements are in the hands of local committees, but a letter to the Lord Mayor of London, or the Mayor of one of the London boroughs, would doubtless receive attention.

### THE SOUTH AFRICAN MEDAL FOR THE NURSE-GARDENER.

We are informed that Nursing Sister Natali Ferriera was presented with the South African medal by Lord Roberts on April 14th. The nurse who has been thus singled out for honour by the Commander-in-Chief is known is South Africa for her successful gardening operations, which have already been mentioned in our columns. Sister Ferriera's experiments at No. 7 General Hospital have been watched with keen interest by the military authorities here, and there is no doubt that they have also excited a spirit of emulation on the spot.

### THE WAR NURSES.

The following nursing sisters arrived from South Africa on board the *Assaye*:—E. G. Eastmead, A.N.S.R., who requires one month's leave and returns to South Africa; R. Moody, A.N.S.R., who is applying to remain at home; E. A. Snape, A.N.S.R., who requires three months' leave and returns to South Africa; and C. Welch, civil nurse, who was locally engaged for the voyage.

### A WOMAN'S ATTACK ON NURSES.

It is as well that since the *Nineteenth Century* has published an attack on hospital nurses by a woman who evidently knows very little about this, the readers of that periodical should have the opportunity of perusing replies from two experts belonging to different sexes. Otherwise, the contribution of Mr. Sydney Holland and Miss Isla Stewart to the May number remind us of the process of breaking a butterfly on the wheel. "The case against hospital nurses," as put by Miss M. F. Johnston, was adequately dealt with in a general manner in our columns on the 12th of last month by a well-known matron; but Mr. Holland naturally takes up the cudgels on behalf of the London Hospital, and Miss Stewart in the interests of St. Bartholomew's. Their task was not difficult, and they discharge it with complete success, quoting definite facts with chapter and verse for them in refutation of Miss Johnston's vague generalities. The truth of the matter is that, as Mr. Holland says, "wholesale accusations prove nothing," and for this reason, as well as for Miss Johnston's obvious lack of qualifications for the part of censor, her charges may be treated with indifference. Both Mr. Holland and Miss Stewart show in detail how entirely she is unacquainted with hospital work and management. We note that on one important point the matron of St. Bartholomew's and Mr. Holland are at issue. Miss Stewart thinks that the position of nurses would be improved by registration under Act of Parliament. Mr. Holland points out, with much force, that "a woman whose technical knowledge and skill are her sole claim to don a nurse's uniform is a very unsatisfactory representative of her profession," and he considers that this is "one reason why it is such a mistake for people to agitate for the registration of nurses." Mr. Holland rightly urges that registration could only deal with the technical qualifications, and would leave the important—we should say the even more important—question of character untouched. He also contends that, "once on the register, it would be almost impossible ever to remove a nurse's name from it except for some gross and criminal act," and "so bad and worthless nurses would be going about hall-marked, and would do still more harm to the public and to their fellow-nurses." This is a sufficient answer to the letter of Dr. Belcher, who advocates in another part of The Hospital the formation of a Trained Nurses' Registration Society.

### THE NURSING DEPARTMENT AT ST. THOMAS' HOSPITAL.

At the general court of the governors of St. Thomas's Hospital last week the report of the treasurer and almoners on the question of accommodation for nurses was adopted. Various schemes have been considered by the Almoners' Committee, who eventually decided in favour of a re-arrangement of the administration block and the adaptation of the present treasurer's house as a home for nurses. The only drawback to this plan is that it involves the sacrifice of the large Governors' Hall, but as the hall is only used on one afternoon in the year, on the occasion of the distribution of prizes, the committee came to the conclusion that its retention was of less importance than the devotion of the space it occupies to more useful hospital purposes. Under the plans prepared by the hospital architect, a good house will be available for the treasurer, and accommodation for about 30 nurses, which will, at all events, materially relieve the pressure that for some time past has interfered with the comfort of the staff. The scheme includes provision for a covered way from the hospital to the home.

### THE PENSION FUND WANTED IN AMERICA.

There is a movement on foot in America for the establishment of a national home for the benefit of graduate nurses who are disabled and unprovided for. This, however, is only a dubious remedy, even if it can be carried into effect. The idea of a number of disabled nurses living together is not altogether felicitous, and the movement also suggests that a large proportion of those who are now in receipt of a fair income are not capable of making any provision for a rainy day. The formation in the United States of an organisation similar to our own Royal National Pension Fund would probably render it unnecessary to proceed with the agitation for a sort of glorified almshouse; and it would have the immense advantage of helping the nurses to help themselves, though hitherto the American nurses have not, as a class, recognised its advantages.

### OPENING FOR PRIVATE NURSES AT RANGOON.

We understand that trained nurses and midwives are badly wanted at Rangoon, and that good pay is offered to them. A correspondent writes:—"My doctor is grumbling at the scarcity of nurses here. He said that if he had a dozen properly trained nurses he could keep them in work all the year round. All nurses get 300 rupees a month with board and lodging, and in infectious cases the payment is continued during the time of quarantine. That is practically £240 a year plus board and longing. My nurse has only had three weeks unemployed in the last year." Lest this statement should be followed by a rush to Rangoon, we think it well to add that the climate is trying, and that minor discomforts have to be faced.

### THE ROYAL SOUTH HANTS NURSES' LEAGUE.

The movement for the erection of a new home for the nurses of the Royal South Hants and Southampton Hospital should receive a considerable impetus from the speeches at the recent public meeting in the Mayor's parlour, at which the Mayor and Colonel Grimston demonstrated the absolute necessity of immediate action. The building in which the nurses are now living is declared to be insanitary, and though the people of Southampton have been somewhat slow to support the hospital as it deserves, they will now, we hope and believe, respond adequately to the appeal to provide the nursing staff with a healthy home. Meanwhile, the nursing staff have shown their enterprise by the issue of the first number of a tastefully got up, and admirably printed *League Journal*. This is to be the occasional organ of the newly-formed Royal South Hants Nurses' League. In the editorial address, the matron, Miss Mollett, explains why the League was formed, and calls attention to the fact that the first annual meeting will be held in June, when it is hoped there may be a large gathering of old friends who will meet one another again, re-visit the old place, and see its alterations and improvements. The "personal paragraphs" are an interesting feature of the publication. We observe that no less than six Royal South Hants nurses have recently relinquished their avocation in order to enter upon the estate of matrimony.

### MATRONS AND TESTIMONIALS.

In spite of some remarks we recently made on the testimonial question, pointing out that to give a testimonial is an act of grace, a correspondent has inquired whether a matron of a general hospital is at liberty to refuse a testimonial to a member of her staff leaving at her own wish in order to take up work elsewhere. We can only repeat that she is at liberty, but in the circumstances cited, we regret that the option, which is properly enjoyed, should have been exercised. The experiences of our correspondent are unusual, but a suggestion that the position would have been improved by intervention on the part of the house committee in such cases, does not strike us as felicitous.

### NEW BEDROOMS AT THE EVELINA HOSPITAL.

At last the nurses at the Evelina Hospital for Sick Children, Southwark Bridge Road, are to have separate bedrooms and pure air, and the hospital will be closed for a period of about six months, in order that the necessary alterations may be carried out. The wards have been emptied—some of the little patients have gone home, others to the seaside or to the country; while the serious cases have been removed to other hospitals. Charwomen have been busy washing and putting away dozens of splints; in the theatre, sister has been polishing, packing, and making an inventory of all the instruments, while in the nurses' room, and in the passages, boxes and other signs of departure were evident towards the close of last week. The matron states that the closing is entirely on account of the nurses. Since 1890 and up to the present the day nurses have been sleeping in the old fever block, close to the workhouse, while the night nurses have had an attic at the top of the main building. It is now intended to take off the roof of the hospital and build twenty-four light and airy new rooms; each nurse will thus be able to have her separate bedroom. Advantage of the closing will be taken to paint the wards, and clean the hospital, and some £6,000 will altogether have to be spent.

### EXAMINATION AT EDMONTON UNION INFIRMARY.

At the last meeting of the Edmonton Board of Guardians the chairman stated that the reports received from the examining medical officer, Mr. John R. Lunn, F.R.C.S., medical superintendent of the Marylebone Infirmary, and their own medical officer, Dr. Benjafield, reflected great credit upon the five nurses who had been trained at the Edmonton Union Infirmary, and on the medical officer and the superintendent of nurses. The result of the examination shows that the total percentage of marks obtained varied from 79·5 to 61·5, and we agree with the chairman, and several other speakers, that the result is a very satisfactory outcome of the first experiment in training by the board. The truism that self-praise is no recommendation does not evidently find favour with one of the guardians, Mr. Cole, who congratulated the doctor, the master, and the nurses upon "having such an excellent

Board of Guardians, who had so generously provided everything that was required for the efficient training of the probationers." The Edmonton guardians are entitled to praise for moving with the times, but under the circumstances the claim for generosity is somewhat unusual.

### DISTRICT NURSING IN THE WEST.

The difficulties which threatened the existence of the Honiton Nursing Association a year ago have been happily overcome, and the report presented at the annual meeting shows how valuable the nurse's services have been to the community. She made 2,088 visits to 119 cases in 46 weeks. It is unfortunate, however, seeing that the nurse now works not only among the poor, but also among those who are able to pay for her services, that there is a financial difficulty. As against £74 in 1900 only £57 was raised last year, and if the Society had not started with £11 in hand there would have been a serious deficit. The example of Dartmouth, where the Queen's nurse established 8½ months since has made over 2,000 visits may be held up as one worthy of emulation. There the year finishes with £50 on deposit and nearly £68 in the treasurer's hands. Honiton, too, might take a useful lesson from Crediton, where Lady Audrey Buller is the Association's president, and a bazaar is being organised in its aid; and from Totnes, whose Association was recently helped to the extent of £14 by amateur theatricals held in the town.

### TAKING CARE OF THE RATEPAYERS' MONEY.

It is not often that protests are made in the supposed interests of ratepayers against the cost of nursing in workhouse infirmaries. But the increase in the poor rate in the parish of Toxteth was condemned in a Liverpool paper, and special complaint was made that there are "too many paid nurses" in Toxteth Workhouse. We infer from the use of the word "paid" that the writer is one of those people who would, if possible, return to the practice, still too frequently indulged in, of pauper attendants "nursing" the sick. Otherwise, as a Toxteth nurse points out, the complaint is quite without justification, for the nursing staff is barely adequate to the general requirements of the wards. It happens, moreover, that the superintendent is very strict in enforcing economy, and that "all the nurses are trained to remember whose money they are using, and cautioned against waste in every respect." The grumbling ratepayer really deserves to be thanked for eliciting this interesting and satisfactory information.

### COTTAGE NURSES IN NORFOLK.

The annual report of the Norfolk District and Cottage Nursing Federation, which was adopted at the third annual meeting, shows that the managers are laudably anxious to improve and extend the training of the nurses. The goal of their ambition is a small central home in a town with a staff of "highly trained nurses" working in the surrounding districts under a sister in charge. But the sub-committee who have been considering the prospects of fulfilment, have reported that at present, owing to the difficulty of older organisations in raising funds, it would not be advisable to start so large a scheme.

### A NEW DEPARTURE BY THE HOLT HOCKLEY ASSOCIATION.

The Holt Hockley Nursing Association are about to open a Central Home at Horsham which will be presided over by a trained lady superintendent, whose nursing career is given in another column. The duties of the superintendent will be to take charge of the Home, give instructions to the nurses, visit them from time to time at their cases, get reports from both doctors and patients. It is also proposed that all candidates shall be tested in the Home before being sent to hospital for training. This Association was founded nearly twenty years ago by Miss Broadwood and the appointment of Miss Bullock as first lady superintendent is an excellent one.

### KENT NURSING INSTITUTION.

The financial position of the Kent Nursing Institution seems to be very satisfactory. The report which was adopted at the annual meeting shows that 2,872 weeks' nursing was done last year; that the 67 nurses employed earned £4,165; that two silver and two bronze medals had been awarded; and that there was a balance of £322 on the right side. An interesting feature of the work is the gratuitous nursing, or nursing on reduced terms. During the twelve months at Malling £140 in fees was remitted, at Tunbridge Wells £28, and at Bromley £28.

### A TIE FOR FIRST PLACE.

At the recent examination of nurses at St. Bartholomew's Hospital, London, Miss May Cooper and Miss Dorothea Mutter tied for first place. Twelve years ago a similar tie occurred. On this occasion the authorities very wisely decided to settle the difficulty by making both nurses gold medallists.

### NURSES' UNION.

An interesting gathering has been organised under the auspices of the members of the Nurses' Union. It is described as a "Sale and Social," and will take place at 26 George Street, Hanover Square, on Friday, June 6, from 3 to 8 p.m., and will be in aid of foreign medical missionary work. Among the speakers will be Miss Wells, formerly a missionary nurse in China; and there will be some excellent music. Miss Dashwood, Shenley, Herts, will be pleased to send any nurse an invitation on application.

### SHORT ITEMS.

Princess Henry of Battenberg has offered the use of her apartments at Kensington Palace for the annual meeting of the Colonial Nursing Association on July 9th.—Lily, Duchess of Marlborough, has promised an annual subscription of £100 to the Royal British Nurses' Association.—A bazaar and fête will be held from May 13th till May 17th at Balls Bridge, Dublin, in aid of the Royal City of Dublin Hospital, to pay off the debt and to defray the cost of the Nursing Home.—About £14 was realised at a jumble sale held on Tuesday, 29th ult., in aid of the Hammersmith and Fulham District Nursing Association.—The marriage at Bombay of Miss Mary Colthurst, who received her training at the Women and Children's Hospital, Cork, under Miss Baxter, to Captain Frank Smith, of the Indian Medical Service, is announced.

### Lectures on Gynæcology for Nurses.

By Robert Jardine, M.D., M.R.C.S., F.F.P. and S.G., F.R.S.E., Senior Physician to the Glasgow Maternity Hospital, Examiner in Midwifery to the University of Glasgow.

### XI.—PELVIC INFLAMMATIONS.

In order that you may form a clear idea of the subject we are now to discuss, it will be necessary to say a few words about the arrangement of the organs in the pelvis. In front we have the bladder, then the uterus with its Fallopian tube and ovary at either side, and lastly the rectum which lies against the back wall of the pelvis to the left side at the upper part. Lining the whole of the interior of the abdominal cavity and covering over all the organs there is a thin serous layer known as the peritoneum. This peritoneum covers over the pelvic organs as well. Thus, if we were to trace it from the front where it lines the anterior abdominal wall, we would find it passed off on to the bladder up over the top of it, then down over the back wall and off this on to the uterus, and up over the top of the uterus, down over its back wall, and then on to the rectum and back wall of the pelvis. At the upper angles of the uterus we have the Fallopian tubes passing out towards the sides of the pelvis, and the peritoneum as it passes over the uterus also goes over the tubes, and thus forms a double fold at each side of the uterus, which extends out to the side of the pelvis. These are what are called the broad ligaments of the uterus. The peritoneum enfolds the tubes in the same way as a sheet does a clothes-line when thrown over it. The lower part of the line is not touched by the sheet and in the same way the lower part of the tube is not covered by peritoneum. The broad ligaments help to keep the uterus slung in the pelvis, but in addition we have several other pairs of ligaments. Where the peritoneum passes from the bladder to the uterus we have a pair of ligaments—the utero-vesical—and between these ligaments there is a pouch—the utero-vesical pouch. Then from the upper angles of the uterus near where the Fallopian tubes start we have the round ligaments of the uterus which extend down between the folds of the broad ligaments and pass out of the abdominal cavity through small canals to be attached under the mons veneris. Behind the uterus we have another pair of ligaments where the peritoneum passes on to the rectum—the posterior ligaments. Between these ligaments we have a pouch which is known as Douglas's pouch. Between the folds of the broad ligament and along all the other ligaments there is a certain amount of what is known as connective tissue. There is a good deal of this tissue at the lower part of the broad ligaments. The vessels and lymphatics lie in this tissue. It is important to keep this tissue in mind as it is in it the swelling occurs in pelvic cellulitis. I have mentioned the ovaries, but we are not concerned with them at present. They are not covered over by the broad ligaments as the tubes are, but they lie on the posterior wall of such broad ligament a little out from the side of the uterus and just below the tubes. They also have their ligaments to keep them in position.

*Pelvic Cellulitis.*—Another name for this condition is pelvic parametritis, but we shall use the term cellulitis, as it is more distinctive. Pelvic cellulitis is the same as cellulitis in any other part of the body, and arises from the same cause, viz., septic absorption. The connective tissue which, we have just seen, lies between the folds of the broad ligaments and along the other ligaments of the uterus, is the part affected. The whole of the connective tissue is not, as a rule, affected, but only that portion of it to which the lymphatics lead from the point where the septic absorption occurs. The part becomes hard and brawny, just as the arm does when a person suffers from a poisoned hand. In the arm, when the hand is poisoned, the glands at the elbow and in the armpit become very tender and swollen. In the pelvis the glands are deeply situated, and out of reach, but they are probably affected in the same way. We have acute and chronic pelvic cellulitis. The former generally follows labour, but it may arise after an injury to the vagina or uterus or an operation on these parts if strict aseptic conditions are not observed. Many severe operations are performed on the uterus and cervix, and the latter is frequently badly injured during delivery, but cellulitis will never arise unless septic absorption takes place. Suppression of menstruation, cold, etc., will never cause it without sepsis being present. As the left side of the cervix is the one most frequently injured during delivery, from the occiput in the vast majority of cases lying to the left side, the left ligaments are the most frequently affected. Septic absorption takes place through the tear, and the lymphatics carry the infection into the cellular tissue which they supply.

*Signs and Symptoms.*—The first indication usually occurs on the second or third day after delivery. The patient feels chilly, and she may have a rigor. Her temperature and pulse rise. The pain may not be very severe at first, but after a while it may become so, especially if the inflammation spreads to the peritoneum, as it is almost sure to do as the peritoneum lies closely over the cellular tissue. There will be tenderness at the side of the uterus. The lochial discharge will be fœtid. At first a vaginal examination will not reveal very much except that the parts are hot and tender, but in a short time a swelling will appear bulging at the side of the uterus. This will be tender, and will very soon become quite hard. The uterus will probably be pushed toward the opposite side. If the cellulitis is extensive, it may be felt in the abdomen lying as a hard mass along Poupart's ligament. It may extend for a considerable distance above the pelvic brim. While one or other side of the uterus is the most usual seat of the swelling, it may be in front or behind. Sometimes it extends right round the cervix, forming a hardened collar-like mass in which the cervix is embedded. The patient's temperature will remain high and her pulse quickened. She may have repeated rigors, especially if suppuration is occurring. The patient will very often lie with the leg of the affected side drawn up so as to relax the muscles in the pelvis and relieve the tension. The bowels may be obstinately constipated, and later on diarrhœa may occur. Micturition may be painful, and there may be retention of urine.

*Course of the Disease.*—In the vast majority of cases the feverishness soon subsides, and the hard mass gradually becomes absorbed. This absorption will take a considerable time. In some cases, however, suppuration occurs in the mass and a pelvic abscess forms. The temperature repeatedly rises, and the patient may have violent rigors. She will not be able to take food, and will become much emaciated. If it is left to itself the abscess will finally point and burst. It may burst into the vagina, the bowel, the bladder, or somewhere through the skin surface. If by any chance it should burst inwardly it would set up septic peritonitis, which would be sure to be fatal. The abscess will not burst for several weeks, seldom less than six, and it may be as many as 12 from the onset.

*Diagnosis.*—The first indication is a rise in temperature and pulse and possibly a rigor. If the case is one after childbirth, it will usually occur on the second or third day. On making a vaginal examination the lochial discharge will be found to be fœtid and a laceration of the cervix will probably be found. At first only heat tenderness and some thickening and perhaps fixing of the cervix may be detected, but by next day a more or less hardened mass may easily be felt. As it increases in size it will bulge down into the vagina and may soon be felt extending up into the abdomen along Poupart's ligament. The patient will lie with the leg of the affected side drawn up. The formation of pus may be diagnosed when there are repeated rigors and progressive emaciation of the patient. If the pus is deep-seated it may be a considerable time before any softening in the mass can be made out, but ultimately fluctuation will become perceptible. The next form of inflammation with which we shall deal, viz., pelvic peritonitis, has many points of resemblance to cellulitis, but we shall point out the differences in our next lecture. It also has to be differentiated from a collection of blood in the pelvic tissues and also occasionally from a fibroid tumour which has grown in between the folds of the broad ligament. The collection of blood usually occurs suddenly, with pain and shock and indications pointing to hæmorrhage. It arises from the bursting of a varicose vein in the broad ligament, or more frequently from the rupture of a tubal pregnancy. High temperatures and rigors will not occur early, but they will occur later if suppuration has taken place. Then there will be a pelvic abscess just as if it had occurred in a mass of cellulitis. With a fibroid there

### LECTURES ON GYNÆCOLOGY FOR NURSES.—*Continued.*

will not be a temperature unless there is some inflammation round it. With cellulitis you will usually be able to make out a source of septic infection and get a history pointing to that.

*Prognosis.*—The majority of cases recover unless great suppuration occurs. Even with suppuration, if proper treatment is adopted recovery should eventually take place, but the patient will probably be ill for a considerable time. Cellulitis is not so apt as peritonitis to leave bad effects, such as adhesions and displacement of the uterus.

*Treatment.*—Preventative treatment consists in the most rigid aseptic precautions in all examinations and operations on the vagina and uterus and also in midwifery work. If due precautions were taken this disease would not arise after operations or confinements. The first point to attend to will be to get the bowels well cleared by giving a saline purge and an enema. If there is retention of urine a catheter must be passed. Obstinate constipation is generally a feature of these cases, and the nurse must see that a motion is secured each morning by a saline purge and an enema. Opiates are very frequently given, but they increase the constipation. The application of hot stapes or poultices over the lower part of the abdomen generally gives considerable relief. A large bran poultice is very useful for this, as it is so light. If the temperature is very high quinine or some other form of antipyretic is sometimes given. Easily digested food must be given frequently, and if the pulse indicates it free stimulation should be resorted to. Vaginal douching twice daily is generally used. The douche should be at about 110° F. and should be given gently. It is soothing, and in the early stages washes out any septic material from the upper part of the vagina, while in the later stages it promotes absorption. Absorption may be assisted by occasional vaginal tampons soaked in glycerine or glycerine and ichthyol. Counter irritants are sometimes applied over the lower part of the abdomen in the form of blisters, or liniment of iodine. When suppuration occurs the pus has to be evacuated, the incision being made through the vagina when possible, but in some cases it has to be made above Pouport's ligament. Free drainage must be secured. The great point in dealing with these cases is to keep up the patient's strength with suitable diet. It is very doubtful if treatment will cut short the disease, as it has to run a pretty definite course, but absorption can be assisted in the way denoted.

*Chronic pelvic cellulitis* has been mentioned. It does not follow the acute form, but arises in connection with suppurative affections of the pelvis. The hard mass will be close to the seat of suppuration and will not be nearly so extensive as in the acute variety.

### "A Distinction with a Difference."

BY AN OCCASIONAL CORRESPONDENT.

"WHAT post do you think of applying for, as you intend to leave?"

It was the matron who spoke, and the capable-looking sister whom she addressed answered without hesitation:

"I shall try and get into a private surgical home."

"A private surgical home! Why so?" The matron's voice expressed the surprise she naturally felt.

Sister Mary had done good work as a nurse, she had been early promoted to the post of sister, and in the common order of things one would have expected that she would continue in the fascinating vortex of hospital life for many years to come and so fit herself for the responsible post of matron. Sister Mary coloured as she answered:

"You see, it is important for me to get as large a salary as I can. I am entirely dependent on what I earn, and I find that my Sister's salary of £27 allows little or nothing for saving."

The matron's face clouded; it was the old cry; she had heard it so often in hospital, and it seemed so unavailing.

"Do you know of a surgical home where you will be paid at a higher rate than in hospital, given that the work expected of you in both be equal?" she asked.

"Oh, yes; I have had several friends who have entered surgical homes and are getting £40 and £45 and the hope of rising."

Sister's voice was brisk and cheerful, contrasting markedly with the tone in which she had spoken of her hospital salary. The matron was silent; she knew as a fact that Sister Mary's statement was true.

"Supposing I applied for a sister's post in another hospital, I should probably not get more than £30 to £35 at the most, and no prospect of rising; and of course I cannot expect a matronship until I have had more experience."

There was a practical ring about sister's words that the matron felt unequal to combat, and her experience taught her that many hospitals were losing their capable senior nurses purely on account of pecuniary reasons. The conversation was interrupted by the entrance of the chairman of the Hospital Committee. He was a man well off in the good things of this world; a hard-headed business man, who expected as much work as he could get out of his subordinates for as little money as he could decently pay them.

"We are likely to lose Sister Mary soon," the matron informed him as sister left the room.

"Indeed, why is that?" he inquired.

"Oh, the usual thing, more money required, and she knows where to get it."

"People don't know when they are well off," he said shortly. The matron looked at him squarely.

"Our salaries in hospital are not excessive for the hard and responsible duties we have to perform." Her voice was somewhat dry in tone.

"We can't afford to make salaries in hospital excessive," he said irritably.

"No; it might be possible to make them adequate," she murmured meditatively. He glanced at her sharply.

"What salary does Sister Mary get?" he asked.

"£27, and she has worked well for the hospital for six years." The matron's voice was expressionless, she had trodden this ground many times.

"Not a bad salary at all; what more does a young woman of her age want?" His knowledge of the demands made on sister's life for the munificent sum of £27 was nil.

"Oh, I don't know." Her voice was still apathetic. "Our cook gets £40, and she has only been with us six months."

"Your cook! That's a very different matter; the patients must have decently cooked food." He was inclined to bluster.

"Yes, I don't find any fault with the amount of cook's wages; the people must have decently cooked food; in my opinion they must also have capable and responsible gentlewomen as heads of the wards to see that they are properly nursed," she said more briskly.

"Well, and haven't you got responsible and capable gentlewomen as sisters?" he asked; he felt somewhat out of his depth with her reasoning.

"Yes, but they won't stop with us at the salaries we offer; they are like cook," she added with a smile; "they want £40; the difference lies in the fact that cook gets what she wants and they don't."

"Tush?" The choleric chairman rose hurriedly, asked if there was anything special to report and then took his leave.

The matron sighed as she resumed her seat. The committee did not have the anxiety of replacing trusted seniors with inexperienced juniors, that was a pleasure left entirely to herself, and the evils resulting from such a practice were usually placed at her door.

### To Nurses.

**WE invite contributions from any of our readers, and shall be glad to pay for "Notes on News from the Nursing World," or for articles describing nursing experiences, or dealing with any nursing question from an original point of view. The minimum payment for contributions is 5s., but we welcome interesting contributions of a column, or a page, in length. It may be added that notices of appointments, entertainments, presentations, and deaths are not paid for, but that we are always glad to receive them. All rejected manuscripts are returned in due course, and all payments for manuscripts used are made as early as possible after the beginning of each quarter**

### Beyond the Seas: The Lunatic Asylum, Jamaica.

BY THE MATRON.

I HAVE a vivid recollection of the difficulty experienced in 1888 in gaining reliable information about Jamaica. I was told in serious earnest that yellow fever and small-pox were always raging; that monkeys and parrots were common objects in the "bush," that tarantulas, centipedes, and scorpions made life miserable; that I should have nothing to do but lie in a hammock and give orders, while a servant fanned away the mosquitoes, as white people were not expected to work; that I should have to drink rum "swizzle" wherever I visited or give dire offence, etc. Jamaica is no longer the *terra incognita* it was. Tourists, journalists, and others who crowd this sunny isle during the winter, have lectured upon and written about it elsewhere, until more is known abroad about us than we know ourselves. A brief account of our institution may interest those readers of The Hospital who are in the ranks of asylum workers.

### Site of the Buildings.

I know many asylums in England and other countries, but none so beautifully situated as ours. Just on the outskirts of dusty Kingston our grounds are bounded on the south by the sea—not the broad ocean, but where the water lies in the graceful curve of the harbour formed by the Palisadoes, as the tongue of land covered with cocoanut palms is called. North and east are the lofty Blue Mountains clothed with verdure. We look our best about July when the poncianas are flowering. Picture an avenue of trees adorned with blossoms something like irises, but of a bright red with centres of white, black, red or yellow; foliage resembling ferns peeps forth amidst the flowers, so that each tree looks like a Brobdignagian bouquet! Overhead is a sky of turquoise blue flecked with fleecy clouds; the purple mountains and green trees form the background; a sea of bluish-green shading to purple under the shadows of the clouds is the foreground, with the golden sunlight everywhere. Is it any wonder that I am enthusiastic over the beauty of our environment? It is lovely at night, too, under the beams of the tropical moon. True the gorgeous colours are almost invisible, but the silvery pathway of light over the waves, and the deep shadows, contrasting with the intense brightness of the moonlight illuminating mountains, trees, and buildings, combine to form a picture whose dainty chastened loveliness may vie with the charms of the day.

### Life in the Asylum.

Of course, life in the asylum is very different from that in a similar English institution. About two-thirds of our patients are negroes, nearly a third are brown, a few coolies, and fewer still whites and Chinese make up the total. General paralysis is rarely seen even amongst the men, and there are few cases of acute suicidal or homicidal insanity. Heredity, ill health, religion of a debased type known as "revival," and the West Indian superstition "obeah" (when the victim believes himself to "have ghosts put upon him," to have "lost his shadow," or to be in other ways under Obi influence), are the chief causes of insanity here. Love, drink, worry, or the high pressure of modern civilisation are not responsible for many of our patients. Since 1888 our population has about doubled. It is now between 860 and 870, but the accommodation has not increased in the same ratio, so we are very over-crowded. To provide for this more adjoining land was bought, and a large extension, on modern lines, costing about £45,000 was built. This is intended for the women, and the men are to occupy all the present asylum. Then came the financial depression from which Jamaica still suffers. Naturally the Government refrains from asking for more money from the over-burdened taxpayers than it is obliged, so this grand range of buildings, almost completed and so much needed, perforce stands empty.

### The Nursing Staff.

Our medical superintendent and two assistant medical officers are from the old country, and have had considerable asylum experience. I am the only other officer from England, where I held similar posts in hospitals and asylums. The nurses are black or coloured natives, mostly illiterate, and otherwise undesirable from an English standpoint. Our ranks are recruited from a lower class than at home. I am sorry to say that respectable, educated Creoles are rarely candidates for employment here. When I came the two head nurses were illiterate women of no use as disciplinarians. One of them once said, "Matron, you don't know my country people yet. Them is not like buckra." (White people.) "Them lie, them tief, them do eberyting that's bad. Why, I would tell you lie myself!" She told the truth then, anyhow. When telling the head night nurse that I had found two nurses asleep she exclaimed, "And I beg them to bear up till you pass through!" Once, on a visit in the middle of the night, I found all the nurses sleeping, but could not find the chief; later, I discovered her in a patient's bed! In 1888 the medical superintendent said, "You see what your materials are; you must make the best of them!" Now, however, they all read and write a little, and are kinder to the patients, take more interest in them, and are more amenable to discipline than formerly. Some read and write well, and have some knowledge of mental and sick nursing. These include the head day nurse, whom I have trained myself, and the head night nurse who has had a certain amount of training at the Kingston Hospital. Still, when we go to the large new asylum I should like two resident English head nurses, with both hospital and Medico-Psychological certificates. Even now we have 440 female patients and 50 nurses and servants. But this dream can never be realised while the colony is so impoverished. One essential difference is that only the doctors, warden, and matron, are resident, and we all board ourselves. Our patients are all in bed before 6 o'clock, when the night staff relieves the day. The consequences of fire in the night, when so few are on duty, do not bear thinking of.

### Amusements and Occupations.

We have no farm or kitchen garden, no band, weekly dance, nor trained choir. There is a nice, convenient, well-lit theatre, but no church. Divine service is held in the dining hall. We average an entertainment monthly, such as theatricals, concerts, or magic-lantern. The expenses also for newspapers, library, cricket, are defrayed from the sale of barrels and other "unconsidered trifles," from charity, and from the earnings of our fishing party. The money realised by the sale of divi-divi (seed-vessels used in tanneries, medicine, etc.) picked up by our women when out walking in the grounds, formerly went to this fund, but goes in these hard times to the revenue. The amateur entertainments, which compare favourably with those at home, are chiefly the results of efforts of my friends and myself. The warden organises the cricket and other sports, and occasionally gets up a concert.

### An Isolated Life.

How miserable, lonely, and homesick I was at first! It was "all work and no play." I knew no one and had no time to return calls. The task of training the nurses seemed too difficult. I could not have borne the life had it not have been for the kindness of the doctors and their wives. I

BEYOND THE SEAS.—*Continued.*

shall never forget Christmas Day, 1889! Someone sent me a branch of berried holly which came from England in the refrigerator of the Royal Mail steamer. How vividly those red berries brought the contrast between that yule-tide and former ones to my mind! I thought of the decorated church, hall, and wards of Macclesfield Asylum; the choral service and Christmas anthem, the patients' fancy ball, the friendly greetings, the carols, and the patients' Christmas Eve teaparty. My lonely dinner was untasted, for I broke down and wept bitterly. But I was never happier in my life than I am now. I like Jamaica, I have made friends, I have plenty of work and recreation too. Occasionally I take a long vacation to "the dear Home-land" and other countries, but I am always glad to return.

### Nursing in an Italian Military Hospital.

BY A SISTER.

During a recent sojourn in Italy I was permitted, by the courtesy of an Italian army surgeon, to visit the military hospital of Florence. The building, which is situated about a mile from the city, was formerly used as a monastery, but is now admirably adapted to the purposes of a hospital. Built in the shape of a quadrangle, with a pleasant garden in the centre, various balconies for the use of convalescent patients, and amidst brilliant sunshine, I could not help thinking it was a most desirable place for an invalid soldier. According to the regulations of the institution, policemen, carabinieri—a higher grade of policeman—in fact all Government officials who require gratuitous treatment are admitted.

### The Wards.

The long narrow wards, containing two rows of beds each, looked clean and comfortable; at the head of each bed was a large square slate indicating the name of the patient, the nature of his disease, and the regiment to which he belonged. In the middle of all the wards was a beautiful flowering plant, a most unusual sight in an Italian hospital. Each ward contains about twenty-six beds and has its special surveillante, or Sister of Charity, of whom there are eleven in all. These Sisters belong to the Order of St. Vincent de Paul, and each one has her separate department assigned to her by the director himself. Among other duties they distribute the wine, superintend repairing of linen, attend to the bathing of patients on admission, arrange and number all the clothing of new patients, give out clothing to be worn in the hospital, supervise the kitchen, cleaning of wards, etc.

### The Nurses.

The nursing is done by orderlies, who are called "Infirmieri," though the ultimate responsibility rests with the Sisters. They do not present the spotless appearance of English nurses, but wear long blouses of some dark washing material and accompany the medical officer on his tour through the wards. They have regular hours off duty, and seem to be a very intelligent class of men. Towards the end of my visit I saw them at supper. Each man had a tin of soup, sundry pieces of meat, and a huge junk of brown bread. After this repast had been disposed of, they one by one repaired to a pump in the yard, and washed up the aforesaid tin. Their sleeping accommodation is elementary, but apparently highly satisfactory. A number of these male nurses are always in readiness to be despatched to the front in time of war.

### The Operation Rooms.

The hospital, which contains about 200 beds, is provided with two operation rooms, one for septic and the other for aseptic cases; stone floors, light-painted walls, operation tables constructed of zinc with movable ends, which can be easily cleansed and disinfected, glass tables for instruments, and a tremendous sterilising apparatus, assured me that the modern Italian surgeon is quite up-to-date *re* Listerian surgery. It is the rule whenever possible, in Italy, to carry every patient requiring surgical attention into a small room off the large ward, where all appliances are kept in constant readiness for immediate use. Certainly this plan saves much labour for the nurse, though it may have disadvantages in other ways.

### First Aid Sacks.

From this hospital is forwarded in time of war, everything needful for the sick and wounded. In a large storeroom I was shown strong wooden cases, duly stamped with the Red Cross of Geneva, and filled with medical appliances; smaller cases for use on the field of battle, viz. "First Aid," sacks containing mattresses, ambulance stretchers of the newest design, arranged so as to form pillow, bed and bedstead combined, baskets with every requisite for an improvised kitchen, cases of instruments for surgical operations, well-supplied medicine chests, and barrels for wine and water.

### Dispensing and Dissecting.

In a large dispensary several assistants were at work, looking very foreign indeed in their queer long blouses. Part of the dispensary was originally the chapel of the building in its monastic days, and frescoes of the Virgin and Saints are still to be seen on the walls, comparatively well preserved. Another large room was devoted to dissecting purposes, and the walls were decorated with sketches of anatomical studies for the benefit of students. On a table under a sheet lay the body of some poor unknown, which the attendant uncovered with the irreverence born of familiarity with such gruesome spectacles.

### Patients' Meals.

The patients' meals are served as follows: ordinary diet begins at 8 A.M. with bread and coffee; at 10.30 soup, bread and meat; and at 3.30 to 4 soup, bread and meat again; and milk in the evening before going to sleep. In the kitchen appetising omelettes were ready for special cases, and sundry chickens were also being prepared. Two or three Sisters with their flappy caps, and voluminous blue robes, showed me the contents of the larder; another exhibited the dark, gloomy chapel where daily prayers were performed, and also the gorgeous robes of the priests in which she evidently took great pride. They seemed very pleased to welcome an English nurse, and asked me anxiously whether I was satisfied with the "pulizia" (cleanliness) of the hospital. Happily I could answer them to their satisfaction. A wholesome smell of carbolic pervaded the atmosphere, and excellent order and discipline appeared to prevail in every part of the building.

### The Roentgen Ray Apparatus.

An interesting object in the hospital was the Roentgen ray apparatus, of which the working was kindly shown by the officer in charge. A carabiniere, who had been wounded in a murderous fray, was summoned for my benefit, and by means of the powerful rays I could distinctly see the fractured bones of his hand, and the fragment of a bullet remaining in his arm. The coins in my purse were likewise distinctly visible, I also saw various photographs of fractures which all had been located by the X-rays.

### Papers for Private Nurses.

BY A SISTER OF A LONDON HOSPITAL.

(*Concluded from page* 54.)

### MEALS WITH SERVANTS.

THIS is a question that sometimes arises in the life of the private nurse, and it is one that calls for the exercise of much tact to arrange to have her meals apart from the servants, when the suggestion happens to be made. There are practically few cases where such an arrangement can be necessary, and a nurse has reason to object to it. Circumstances alter cases we know, and nurses and servants must be looked upon from the individual point of view and not as classes. In one case, for instance, the nurse will be a lady by birth or education, or both, while perhaps the special set of servants amongst whom she may be expected to take her meals may be coarse and vulgar, and the behaviour, and especially the conversation, would be a trial to her every time she sat down to a meal, and a far greater strain to her nerves and temper than the most trying patient could ever be. On the other hand, the nurse herself might be unrefined, fond of gossip, and inclined to prefer any kind of society to a "dull" meal by herself, and in some cases old and valued personal servants from long intercourse with persons of culture and refinement would be essentially above the unrefined though trained nurse. So that it is scarcely from the point of social difference that any objection should be made.

### LARGE HOUSEHOLDS.

It is seldom in any but very large households that the difficulty presents itself, and by no means as a rule then. Where there are two or three servants only, a nurse is mostly invited to the family table, as it saves the extra work of carrying meals up and down stairs. In the larger households it is generally the housekeeper who arranges for the nurses' meals, and she, because a nurse is a working woman, will place her in her own mind with the upper servants and will very likely think she will enjoy a gossip over her meals as a welcome change from the monotony of the sick-room. But a gently worded request—"Would you mind having my meals sent up to my room," or the nearest room to the patient's which is available, as the case may be, "I like to be within easy reach of the patient"—always a good reason—since housekeepers' rooms are usually very far removed from the family bedrooms, will generally meet with a ready "Certainly nurse if you prefer it, but it will be very dull for you," or some similar speech. But there must be no hint of the fact that the nurse does not care for the society of the steward's room. It would be fatal to her after comfort. Where younger members of the family are the patients, the schoolroom or the nursery will often be suggested, and to this there can be no possible objection. It is a very nice arrangement. Heads of large nurseries are frequently ladies nowadays, and if not, are usually kind and sensible women, who will be far less likely to resent the help of the trained nurse or to be jealous of her, if the nurse does not hold herself superior to her, and make much of the distinction between her hospital training, and the nursery training of the other.

### A "BIRD OF PASSAGE."

A nurse is but a "bird of passage," not a member of the family, or household, nor a visitor, and the way she is treated for the time-being entirely depends upon the people amongst whom she lives temporarily. But whether she be treated by them as an honoured guest, as a member of the family, as an upper servant, or, as in a few cases happens, is considered entirely beneath their notice at all, does not in the least affect her social position. It is essential to cultivate the power of adapting herself to all kinds of people; and should she unfortunately find herself amid uncomfortable surroundings and with uncongenial people, she has at least the consolation of knowing that her post is but a temporary one, and the next case may make up for it. Tact and consideration for others can do much to help her over difficulties. Servants are very quick to notice those who are thoughtful for them, and it often happens that where others are not too kind, they will perform many little kindnesses when they find that nurse tries to save them trouble.

### THE QUESTION OF GOSSIP.

In large households a nurse can never be too much on her guard against the love of gossip which exists amongst "upper" servants, and herein lies the greatest of all objections to the nurse taking her meals with them. What is said and done by "the family" is of intense interest to them, and they will evince the greatest curiosity as to what goes on in the sick-room. A nurse who possesses the true spirit for her work will reverence the intimate knowledge she must of necessity gain concerning her patient, and will find the perpetual questioning one of the trials that beset her; a trial which, putting aside all other considerations, will be quite enough to make her seek privacy in the times she spends away from the sick-room. On the other hand, if the nurse should chance to be less refined, willing to please by retailing bits of gossip, tales of her patient or of other patients, then there is the greater reason that she should have little intercourse with the household. There is of course a very great deal that may be told and much anxiety spared in the case, especially of old servants, who find it hard to see a stranger waiting upon those who in a general way depend upon them for attention to their personal needs. Those nurses who on entering a household remember that it is their privilege "not to be ministered unto, but to minister" will seldom have cause to complain that they have been "treated as if they were servants," for all will be ready to acknowledge the help and comfort they have given in a time of sorrow and distress.

### Presentations.

ST. OLAVE'S UNION.—A handsome cruet and salad bowl has been presented by the matron of St. Olave's Workhouse, Ladywell, on behalf of the officers, to Charge-nurse Baker, on the occasion of her marriage.

MONKWEARMOUTH AND SOUTHWICK HOSPITAL, SUNDERLAND.—Miss E. F. Deakin has recently resigned the post of matron to this hospital, which she has held for the last five years. During her stay she has earned the respect and esteem of all connected with the institution on account of the excellent work she has done. Prior to leaving, she was the recipient of an oak tea-tray from the honorary medical staff, afternoon tea-cups and saucers from the house surgeon and nursing staff, in addition to other gifts from the secretary, several members of the committee, and two old house surgeons still resident in the town. The good wishes of all her late colleagues accompany Miss Deakin in the new sphere of labour to which she has been appointed, namely, the matroncy of the Durham County Hospital.

### Novelties for Nurses.

BY OUR SHOPPING CORRESPONDENT.

### THE "PES DUPLEX" DURABLE HOSE.

MESSRS. WHITE, of Loughborough, have manufactured a stocking which especially recommends itself to nurses. Both the ankle and entire foot are doubled by a new process which greatly strengthens all parts of the stocking coming in contact with shoe or boot without appreciably adding to the bulk, or interfering in the least with comfort. The stockings bear the trade mark "Pes Duplex," and can be bought either ribbed or plain. The quality is of the best.

### Everybody's Opinion.

[Correspondence on all subjects is invited, but we cannot in any way be responsible for the opinions expressed by our correspondents. No communication can be entertained if the name and address of the correspondent are not given as a guarantee of good faith, but not necessarily for publication. All correspondents should write on one side of the paper only.]

### TRAINED NURSES' REGISTRATION SOCIETY.

"Henry Belcher, M.D." (retired), 28 Cromwell Road, Brighton, writes: As a medical practitioner of many years' experience it occurs to me that the time has now arrived to place trained nurses on a firmer footing affording them, as well as the general public, protection against impositions. To meet this difficulty I suggest that a registration society should be formed under Government supervision to be called "The Trained and Registered Nurses' Society," adopting a universal uniform or badge, with the power to impose a penalty or fine on anyone assuming the same unjustly. I shall feel very grateful for any hints or suggestions as to how to proceed in this important scheme, which will render tangible service to the most noble and unselfish portion of our fellow creatures.

### THE QUESTION OF STANDING.

"K. T." writes: A. G. in speaking of the prevalence of flat foot amongst nurses suggests that the undermanning of the staff is the main source of this. I would mention another cause, which an increase in the number of nurses would not remove. In how many hospitals is all the sitting down that is possible encouraged? I would also mention another of the minor diseases which nurses suffer from and never speak of. After three years' hospital work how many nurses are there who do not suffer from varicose veins and burst capillary veins all over their legs? and do the sisters, doctors, or matrons hear of it? No! nurses keep all these things strictly to themselves, for to take steps to prevent them would be, they believe, to hinder their chances of promotion. Now, if 50 out of 100 nurses are truly suffering in this way, is it because 50 women are so carelessly chosen that they are particularly liable to varicose veins, or is it that the work that is done and the material which does it is strongly at variance? A nurse being on duty from 7.30 A.M. to 1 P.M., how much sitting is she permitted to take during that time? After thirty minutes or less for dinner, the morning of standing is followed by from four to six hours at least in the afternoon, and one hour at least of standing must be done daily in connection with the nurse's own affairs—dressing, shopping, taking the air, amusements, etc. Do physiological facts show that women are so constructed that they are able to stand for so many hours daily, Sunday and week-day, for three years? Given a larger number of nurses, how many sisters would dare to suggest that they should sit down in the face of things as they are? Would the matron, in face of constant visits from governors, etc., be as brave as to encourage restful composure amongst her nurses; or would she not prefer that active stirring about should be the more noticeable thing? Who, then, is to fight this fight for less standing for our nurses?

### MALE NURSES IN GENERAL HOSPITALS AND PRIVATE WORK.

"A Hospital Certificated Male Nurse" writes: I was glad to see a reply to the "Note" of April 12th as regards male nurses. I think that the comment on those of us who have had hospital training and hold certificates is most unfair. I feel sure there are some who not only follow nursing for a living, but as nurses endeavour to forward to the best of our ability the progress of the patient under our charge. As regards the case spoken of in the issue of April 12th, as to the attraction of men to become nurses, I think after six years' hospital and infirmary and three years' private experience, I can safely say that there is as hard work in the sphere of nursing as in other fields of employment which are open to men in the labour market at the present time. I myself was trained under the supervision of a medical superintendent, matron and sisters, who each did all they could to teach us as probationers. As regards general hospital training, I feel sure that there are a good many more besides myself who would have welcomed the chance of entering a general hospital to obtain such training.

"C. E. S." writes: As a male nurse I should like to thank "Medicus" for his letter speaking so well for our sex. I have been reading the letters since they first commenced on Male Nurses, thinking that some medical man would at least speak up for us, as I know of a great many who think highly of us. It is quite true, as Mr. Guttridge says, that people reading advertisements in the daily papers often employ men who have been discharged from one of the co-operations in London. The co-operation I am employed by is very strict as regards character and proficiency in nursing, also as to sobriety. Each man must hold a total abstinence card, which is, I think, a great help to all nurses. I am sure that a great number of our men, if not all, take the greatest interest in their work, and when I say that we are in constant employment, and can seldom get a holiday, I think it will tend to show that at least the general public and most of the medical profession, if not all, approve of the male nurse.

"Thistle" writes: In answer to the letters of W. Gutteridge and "Medicus" I would like to say a few words anent the male nurse. Certainly he is a badly-needed individual, nearly as much so as the lady doctor; but the cases are very exceptional in which a trained woman cannot do all that is necessary for a sick man without in any way losing either his respect or her own. Before the trained male nurse can hope to take the place of his female competitor his training in nursing the sick should be equal to hers. As one of the "old staff" of the Derby Borough Asylum I would be the last to decry the excellent training to be found there; the medical superintendent of that institution has spared no pains in raising the standard of mental nursing among his staff as high as possible, and those of us who have had the pleasure of working under him honour him for the good work he has done and still continues to do in that direction, and we are proud when he gets even a small portion of that appreciation so justly his due. At the same time, I fancy that Dr. Macphail would agree with me, that his first aim is to train the best possible mental nurses, his second to have those nurses trained to nurse skilfully any cases of sickness likely to come under their care in the ordinary exercise of their avocation. An asylum is pre-eminently a "hospital for the insane," and does not claim to be a training school for general nursing. Nowadays a lot of nonsense is talked about the coarsening effect certain nursing details may have on the female members of the nursing profession; surely, if this be so, the rule holds equally good for the medical profession and the male sex; and yet what profession is more respected? and respected justly also! Perhaps, though, this only proves the superiority of the "male mind." At all events, as long as men allow their wives and daughters to be attended by doctors of the opposite sex, just so long should they have the decency to hold their tongues on the "coarsening" subject, and, like Brer Rabbit, "say nuffin'."

### SLEEPING IN A PATIENT'S ROOM.

"River Thames" writes: I was truly glad to see the letter of "Medicus," and the letters of "R. J.," "Fuzzy Wuzzy," in reply. I have often wondered why no strong line has been taken by nurses on the subject of the male catheter. I was trained at one of the largest London hospitals, where, of course, such a thing as nurses attending what we called "screen cases" was unheard of. Some years later, when I became matron of a small hospital, I was astonished to find the senior nurse there was accustomed to pass the male catheter, and that the doctor expected her to do so. The new nurses objected to such a practice, and when I spoke to the doctor on the subject, and said that my sympathy was entirely with the nurses, and that I thought they were perfectly right to object, he was quite surprised, and seemed to think that all nurses were accustomed to attend screen cases as a matter of course. I think if nurses when they go out into the world would make a firm but respectful objection to such demands on the part of doctors and patients' friends the doctors would most readily respect the nurses' feelings, and the friends would gradually recognise that there are certain things which a nurse's self-respect forbids her to do, and which she ought not to be asked to do.

"Alexis" writes: May I offer to "Medicus" my earnest thanks for his letter. Would we had more members of his profession with his views on the subjects of his letter. I shall go even further than he, and say that passing a male catheter is not alone indecent, but wrong, very wrong. I have been nursing for two years, and cannot understand any woman doing it. As I write I fancy I hear some nurses say, "What is to be done ?" Refuse, simply and absolutely, to do so, and by so doing win the respect of every refined-minded man and woman. I have met many doctors during my nursing career, and not even once has it been suggested I should do so. In one case where it was necessary it should be done, and where there were two of us, the doctor taught the patient's son, and I am sure that in all cases a way can be found to avoid it, if, as "Medicus" says, the nurses and doctors are only high-minded enough. I knew a gentleman once who had been a patient in a large hospital where the catheter was passed on him by a nurse. His sister's ambition was to enter the profession. When he returned home his words to her were, "I would rather see you dead." I am quite sure it is thus the majority of men feel, who have had his experience, and, thank God, there are such men in the world. I know there are coarse doctors who think it is a nurse's duty to do it, but none coarse enough to be surprised at her refusing. It is solely and entirely the fault of those who do it. I feel very sorry indeed for any woman who thinks that not passing a male catheter is showing want of sympathy. May I, and every other woman, be without sympathy if it is to be expressed so. With regard to sleeping in a male patient's room, I can only say that the nurse who does so cannot expect respect from anyone, and certainly has none for herself, and, really considering many things, one ceases to wonder when she hears nurses spoken of as they often are. Let us, as women, join together and put down these practices which are a disgrace to the profession.

"Bodie" writes: May I be permitted to give a little of my experience on above subject, having been private nursing for ten years ? One case of paralysis, a man aged 34, perfectly helpless. I slept in his room nearly two years. The "most exceptional circumstance" (that of passing catheter) did not arise; but had it done so I should not have dreamt of being "squeamish"; as it was, I washed my patient twice daily from head to toe—that to me is just the same as bathing a male. My next case of paralysis was a man aged 87. I slept in his room 16 months. Then I had a case of tuberculosis of the rectum, with any amount of dressings, etc., necessitating my sleeping in the patient's room, a male, 34. Another case was that of a male of 80, a catheter case. I also slept in his room, as the catheter was passed every six hours. Yet, from my own personal experience, I can unblushingly say that up to the present moment my feelings have never been outraged; any nurse whose feelings are so squeamish had far better not have entered our noble profession. Nothing that I can ever do for a patient (especially a helpless male) is to me degrading. As a man thinketh so he is. This I presume applies to nurses. Someone may suggest a male nurse. "Rubbish." You will never get a man to nurse so successfully as a woman, neither will you get a lady doctor to equal a medical man. I have pitied many a nurse, fresh from three years' hospital training, stranded, in emergency, with a male catheter case. Why should this be ? I myself have seen a doctor pass a female catheter. Why is not the latter as absolutely indecent as the former ? I have always been treated with respect and consideration, and found all my male patients (and they have been many) gentlemen in the highest sense; but, on the other hand, I have known cases where a male has not been quite a gentleman. I, too, blush to say that all nurses are not what we ought to be. In spite of "Medicus'" letter I still go on with this "utterly degrading practice" (passing the catheter).

### Appointments.

[No charge is made for announcements under this head, and we are always glad to receive, and publish, appointments. But it is essential that in all cases the school of training should be given.]

Belper Workhouse Hospital.—Miss Catherine Edith Claridge has been appointed staff nurse. She was trained at Chorlton Union Hospital, and has since been sister at Christ's Hospital, Sherburn.

Cardiff Union Hospital.—Miss M. N. Hayes has been appointed charge nurse. She was trained at Cardiff Union Workhouse Hospital, and has since done private nursing at Shrewsbury.

City Hospital, Walker Gate, Newcastle-upon-Tyne.—Miss Charlotte de la Fontaine has been appointed night superintendent in the place of Miss Swallow, who withdrew on account of her mother's illness. She was trained for three years at the Royal Infirmary, Glasgow, where she was afterwards charge nurse two years. She has been district nurse under the Notts County Council, night superintendent at the new Infirmary, Isleworth, and assistant matron Kent County Asylum, near Canterbury. She holds the L.O.S. certificate.

Ecclesall Bierlow Infirmary, Sheffield.—Miss M. Cooper has been appointed charge nurse. She was trained at Chorlton Union Hospital.

Frimley Urban District Isolation Hospital.—Miss C. Beeton has been appointed matron. She was trained at Liverpool Royal Infirmary, where she was afterwards staff nurse. She has also been charge nurse at Homerton Fever Hospital.

Hackney Union Infirmary.—Miss Emilie L. Foskett has been appointed assistant matron. She was trained for three years at the East Sussex Hospital, and has since held the appointments of sister at the Leeds Union Infirmary, and assistant matron at the Stirling District Asylum, Larbert. She has also been assistant matron at the Rescue Home, Belfast.

Hospital for Infectious Diseases, Gateshead.—Miss Wilson has been appointed matron. She was trained at Gateshead Fever Hospital, and has since been assistant nurse at Chorley Fever Hospital, and assistant nurse at Sunderland Fever Hospital.

Rhondda Urban District Isolation Hospital.—Miss Jessie McGregor has been appointed staff nurse. She was trained at the Sanatorium, Cardiff, where she has since held the post of assistant and staff nurse.

Royal Halifax Infirmary.—Miss Gertrude Aitchison has been appointed sister. She was trained at Paisley Infirmary, and has since been nurse at a surgical home, staff nurse at the Sick Children's Hospital, Edinburgh, and staff nurse at Oldham Infirmary.

St. Olave's Union Infirmary, Rotherhithe.—Miss Lydia Kathleen Lindo Craven has been appointed charge nurse. She was trained at County Clare Hospital, where she was afterwards staff nurse. She has since been charge nurse at Highgate Infirmary, night charge nurse at Bethnal Green Workhouse Infirmary, and ward charge nurse at Wandsworth and Clapham Workhouse Infirmary.

The Fever Hospital, Liverpool North. — Miss Gweullian Evans has been appointed charge nurse. She was trained for two years at the Middlesbrough Fever Hospital, and subsequently for three years at the Leeds Union Infirmary.

The Nurses' Hostel, Horsham. — Miss Amy Bullock has been appointed lady superintendent. She was trained for one year at All Hallows' Hospital, Ditchingham, three years at the Grosvenor Hospital for Women and Children, Westminster, and three years at the London Hospital. She has been temporary nurse matron at the Infirmary of St. Nicholas Industrial School, Manor Park, assistant matron at St. Mary's Infirmary, Islington, and home sister at the Royal Hants County Hospital, Winchester.

Wrexham Joint Fever Hospital.—Miss Myra Bushnell has been appointed matron. She was trained at the Royal Infirmary, Newcastle-on-Tyne, and Brook Fever Hospital, Shooter's Hill, London, where she has since been charge nurse.

### Echoes from the Outside World.

### Movements of Royalty.

THE King and Queen, the Prince and Princess of Wales, and other members of the Royal Family have resumed their positions as patrons of the Royal Military Benevolent Fund, and Lord Roberts, Colonel Sir E. W. D. Ward, K.C.B., and Sir Henry Burdett, K.C.B., have become trustees. The object of this Fund is to give annuities up to £40 to ladies in necessitous circumstances, exclusively the widows or unmarried daughters of officers in the Army or Royal Marines. At present there is only sufficient money to pay the annuities of those already on the list. Seventy applicants are awaiting an increase of the receipts before their poverty can be relieved. Their need will specially appeal to those who have suffered nearly from the present war, or who have particular cause for thankfulness that their own relatives have been preserved.

On Saturday the Prince and Princess of Wales visited the Crystal Palace in order to be present at the annual festival of the London Diocesan Juvenile Branch of the Church of England Temperance Society. The Princess distributed the prizes to the successful children, and the Bishop of London, in proposing a vote of thanks to their Royal Highnesses, said that the Society had adopted as its rallying cry "Wake up," the exhortation given by the Prince of Wales to the English people, at the Guildhall last year.

The Duke and Duchess of Connaught received a hearty ovation from the citizens of Wolverhampton when they visited that town on Thursday last week in order to open the exhibition. The date fixed for the ceremony, May 1, happened to be the Duke's birthday, and Lord Dartmouth in his speech was able to offer the Royal visitor his congratulations. His Royal Highness made a felicitous allusion to the "immense trouble and immense expense" entailed by the exhibition, and went on to hope that "the endeavours which have been made by so many men who take a deep interest in all that concerns the trade and energy of our country will be repaid by the exhibition proving a centre not only in that part of the kingdom, but throughout the whole of it, and through other countries too." The present is the third exhibition of its kind held in Wolverhampton, but it is on a far larger scale than either of its predecessors.

### The Royal Academy Banquet.

ON Saturday evening the Prince of Wales was present at the Royal Academy banquet. After the toast of the health of "The King" had been enthusiastically received, the President gave that of "The Queen, the Prince and Princess of Wales, and the other members of the Royal Family," to which the Heir-Apparent replied. At the outset he made an allusion to the blank caused by the absence of the King, who for 39 years had almost invariably attended the dinner, and assured the company that his Majesty would continue to display the same interest in the prosperity of the Academy in the future that he had shown in the past. This interest, it may be mentioned, was evinced in a striking manner on the day of the private view, when the King came up from Newmarket especially in order to see the pictures, and spent two hours with the Queen in the galleries of Burlington House. The Prince of Wales, at the close of a most interesting speech, in which he suggested that facilities should be offered to students in Australia, who now completed their studies in Paris, to finish them in London instead, announced that copies of the portrait by Mr. Luke Fildes of the King, are to be made for the Embassies and Government houses in India and the Colonies.

### Foreign.

THERE was alarming news on Monday respecting the Queen of Holland. At six o'clock on Sunday evening her Majesty was prematurely confined, and at eleven her condition was described as critical. At seven the next morning it was intimated that, "the Queen is still alive, but her condition is so serious that not one of the doctors has left the Palace, and the Queen-mother and the Prince Consort did not go to bed. The accouchement was long and difficult." Later in the morning a more reassuring bulletin was issued, and at two in the afternoon it was announced that "the condition of the Queen is satisfactory up to the present." Since then, however, rather less favourable bulletins have been issued.

The Princess Radziwill, the Sclavonic adventuress who was last week sentenced to undergo two years confinement in a house of correction by the Chief Justice of Cape Colony, for fraud and forgery, was at one time a person of note in Europe. During the famous Congress of Berlin, in 1878, she made the acquaintance of Lord Salisbury, and she has been received in the best society in the British capital. When she arrived in South Africa she settled in Cape Colony, and soon became a frequent visitor at Groote Schuur, the residence of the late Mr. Cecil Rhodes. The Chief Justice, in addressing the princess, after the jury had returned a verdict of guilty, referred to her birth, education, and position in society, and mentioned her attempt to throw the guilt on an innocent woman, and the bribing of a lad in the post office to forge the Hawkesley cable, as dark features of the case. But for the fact that she is in bad health, she would have been condemned to hard labour as well as detention.

### Art and Literature.

THE Exhibition at the New Gallery is interesting, but there are only a few pictures which call for special mention. Foremost amongst these are the two canvases by the veteran artist, Mr. Watts. The most important is an allegorical subject "Love Steering the Boat of Humanity." The picture is full of life and instinct with poetry, a characteristic which is also noticeable in the charming little wooded landscape, by the same artist. Mr. Sargent is to the fore with striking portraits of three children and their three dogs, grouped together on a sofa so as to form a marvellous mass of red, white, and black, and Mr. Shannon has a remarkable sketch of the Marquis of Granby's little daughter, Lady Diana. Sir James Linton's "Madonna" shows much careful detail, and Mr. Nettleship's great black bear, crunching the bone of an animal in the evening twilight, is almost appallingly realistic. Mr. Llewellyn contributes a portrait of Mrs. R. C. Priestley, which should increase his reputation, and Lord Milner's portrait, painted by Mr. Hugh Glazebrook, will be much discussed. The collection of specimens of Japanese art, in the entrance hall, is well worth a study. Some of the gold lacquer is particularly fine.

The death of Mr. Bret Harte at Camberley, Surrey, was announced on Tuesday. Bret Harte, who was in his sixty-third year, was one of the most popular of American humourists and a poet of considerable repute. From 1880 to 1885 he was U.S. Consul at Glasgow, and he has since resided chiefly in this country. Among other works, "The Heathen Chinee," "The Luck of Roaring Camp," "Gabriel Conroy," and "Three Partners," are popular here; and of his poems, "Plain Language from Truthful James," published in 1870, secured him immediate fame on both sides of the Atlantic. His last work, published a few weeks ago, was "On the Old Trail."

### Amusements.

THERE have recently been added to the attractions at the Zoological Gardens three penguins, of which two, brightly coloured with black, white, and yellow, are placed near the seals. Other additions to the collection are a female condor and two Nylghaies. According to the annual report just issued, the number of animals living in the gardens at the end of last year was 2,922, of which 789 were mammals, including the fine giraffe, 1,575 birds, and 558 reptiles and batrachians.

The New Palace Steamers, Limited, will start their popular steamers for the season on Saturday, May 17th, in time for the Whitsuntide holidays, when the *Royal Sovereign* and *Koh-i-noor* will sail to Southend, Margate, and Ramsgate. *La Marguerite* will begin her trips to Margate, Boulogne, Calais, and Ostend, on July 1st. A special trip is to be made by the *Marguerite* to Southampton on Friday, June 27th. During the winter the steamers have been thoroughly overhauled and improvements made.

### A Book and its Story.

### WALTER BESANT.*

THE autobiography of Sir Walter Besant is primarily a record of the successful career of a conscientious painstaking literary man, who began life as a master at Leamington College, who had aspirations to become a poet, who "would rather have been Tennyson than any man of the century," and who finally settled down to writing novels, whose limpid prose is unsuggestive of the care and rigorous revision that every written line received before it appeared in print. The book as it stands will be of first interest to those who are interested in literature as a profession. Everyone may not start with the mental equipment of the author, who was from childhood an intellectual leader; he was also by nature a student, a reader, and he became later a distinguished scholar, but the patient care bestowed upon the production of all that bore his name is within the reach of the humblest aspirant to literary distinction. He writes with enthusiasm of everything connected with the art he loved so well, he was intolerant of all that lowered in any way the profession of letters; and his impatience of critics, about which so much has been written, arose not from any personal feeling, but from knowledge of the dignity of literature, and "of the important part that novels play in the education and development of the people." Sir Walter Besant was born in Portsmouth, within sight and sound of the great dockyard whose busy voice to passing strangers and visitors is one confusing deafening noise; but to those who, like himself, lived close by that busy centre of industry, and had imagination, every stroke had its suggestive sound, forming a harmonious and pleasing whole. There were Polish exiles in Portsmouth with whom he made friends, and one of his first novels contains minute and picturesque descriptions of old Portsmouth and its environs, and of Ladislas Paloski, a Pole, who tells the story. Little mention is made of his father, who was apparently a studious, retiring man, whose chief delight was in the quiet pleasures of gardening and books, and the occasional exchange of visits with congenial friends. Of his mother he writes with affectionate fervour. "She was the cleverest woman I have ever known; the quickest witted; the surest and safest in her judgments; the most prophetic for those she loved; the most far-seeing." She had a genius for household management and the bringing up of children; she was essentially a woman and a mother, as the affection with which Sir Walter recalls his early home life, of which she was the sun and centre, testifies. Not a woman of education, but a woman of character and imagination. She early aroused the faculty of imaginative narrative when, in the evening twilight, she gathered the children around her and told them stories of the New Forest, where she had lived as a child—stories that aroused the imagination of her third son, Walter, till, gazing into the red coals, he saw, as in a procession, the figures of the story pass before him and act their parts between the bars. "My mother gave me such imaginative powers as have enabled me to play my part as a novelist; it is my inheritance from her. To others of her children she gave other gifts; to one the mathematical mind, to another a marvellous memory and grasp of figures quite remarkable." Her eldest son, Dr. W. H. Besant, was quite brilliantly gifted. He took everything before him at school and college, and became Senior Wrangler and first Smith's prizeman. Sir Walter entered King's College, London, in 1854, at the age of 18, and the following year he went to Christ Church, Cambridge. Here he was fellow student with many notable men who have risen to distinction in divinity, science, literature, and the law. Sir Walter took the gold medal of the year for an English essay, he writes, "was bracketed with C. Stuart Calverly, poet and scholar, incomparably the most brilliant, the finest scholar, and the most remarkable man from every point of view of his time. For a freshman of 19 to be bracketed equal in an English essay with the most brilliant scholar of his time was too much to be expected. I have never since experienced half the joy at any success which I felt on that occasion." Scholar, exhibitioner, and prizeman of his college, and obtaining a tolerably high place among the wranglers of his year, Sir Walter yet failed to attain to the higher distinctions which, with his unusual abilities, his friends aspired to for him; for, a few years after leaving college, he took up tutoring, first at Leamington College, then at Mauritius, where he was attached to the college as a professor. He was offered the rectorship upon the retirement of the rector of the college, but his health had been affected by the climate and he severed his connection with the Ile de France. His first novel, written at this period, was rejected by the publishers, with the comment—"Has promise." A series of published essays on "Early French Poetry" met with some success. In 1868 he became secretary to the Palestine Exploration Fund, a lucrative post which enabled him to carry on his literary work at the same time. Ten years later he married. He suffered from shortness of sight, and he refers more than once to the difficulty this caused in not being able to see or enter into the enjoyments which otherwise would have given him keen delight. Perhaps no more successful instance of literary collaboration can be cited than that of Besant and Rice. Rice was the editor of "Once a Week," and Besant's first interview with him was on the occasion of his making a complaint at the way in which an article of his, "a pretty little thing which found many friends," entitled "Titania's Farewell," had appeared in its columns, badly printed and without corrections. Rice apologised, explained that he had only just taken over the paper and seeing the article in type and approving of it had had it printed. This interview, 1869, was the beginning of a friendship which lasted till the death of Rice, thirteen years later. The collaboration was in every way a harmonious and successful one, and Sir Walter generously acknowledges his indebtedness to Mr. Rice with regrets that he did not live long enough to establish a literary reputation which could be maintained alone, as in his own case. Sir Walter Besant's sympathy with the overworked and underpaid classes is too well known to need comment. He instituted the Home Arts Association, which now includes over five hundred schools in different parts of the country. The work is done at evening schools and attended by the artisan class. The Women's Bureau of Work is also another of his philanthropic schemes. He did not live to complete his great work "The Survey of London" which was written in the intervals of fiction production. The survey he speaks of as "a part, a good part, of my life's work." The Autobiography claims the indulgence of the reader from the fact that it has not received that careful revision which Sir Walter gave to all his written work. He died before it was prepared for the press. Mr. Squire Spriggs in a prefatory note says, "For my own part I am sure that he would have improved the autobiography in certain directions . . . and I am equally certain that the work as it stands must have a useful, nay, a noble influence." In the following striking words he sums up the personality of Sir Walter. "A scholar who was never a pedant, a beautiful dreamer who was a practical teacher, a modest and sincere man, who teaches with conviction a brave scheme of life."

* "The Autobiography of Sir Walter Besant." Price 6s. net. Publishers: Hutchinson and Co.

### For Reading to the Sick.

ASCENSIONTIDE.

O Lord Almighty, Who hast formed us weak,
With us whom Thou has formed deal fatherly ;
Be found of us whom Thou has deigned to seek,
Be found that we the more may seek for Thee ;
Lord, speak and grant us ears to hear Thee speak ;
Lord, come to us and grant us eyes to see ;
Lord, make us meek, for Thou Thyself art meek ;
Lord, Thou art Love, fill us with charity.
O Thou the Life of living and of dead,
Who givest more the more Thyself hast given,
Suffice us as Thy saints Thou has sufficed ;
That beautified, replenished, comforted,
Still gazing off from earth and up at heaven
We may pursue Thy steps, Lord Jesus Christ.

*C. Rossetti.*

Genuine belief is ever followed by corresponding practice. The Ascension of our Lord is a truth which should beget a spirit of unworldliness and detachment in His disciples. This is the fruit which satisfactorily proves our belief in the Ascension to be a genuine belief. The Ascension of Jesus Christ reminds us that we are but "sojourners and pilgrims," but "strangers and pilgrims on the earth, seeking after a country of our own," who "desire a better country, that is, a heavenly," to whom this bustling world possesses but a transitory interest. Since the Ascension our true home is above. The Ascension sets before us a life, which is higher than the world, and that which belongs to it ; and constrains us to "seek the things that are above, where Christ is, seated on the right hand of God," and "to set our mind on the things that are above, not on the things that are upon the earth."

In pressing this particular view of the result, or practical outcome of belief in the truth of the Ascension of our Lord Jesus Christ, we are following the teaching of Holy Scripture, and the guidance of the Church as expressed in the collect for Ascension Day.—*V. Staley.*

Grant we beseech Thee, Almighty God, that like as we do believe Thy Only-begotten Son our Lord Jesus Christ to have ascended into the heavens, so may we in heart and mind thither ascend, and with Him continually dwell. Who liveth and reigneth with Thee and the Holy Ghost, one God, world without end.

Name Him, brothers, name Him,
With love as strong as death,
But humbly and with wonder,
And with bated breath :
He is God the Saviour,
He is Christ the Lord,
Ever to be worshipped,
Trusted, and adored.

In your hearts enthrone Him !
There let Him subdue
All that is not holy,
All that is not true.
Crown Him as your Captain,
In temptation's hour ;
Let His Will enfold you
In its light and power.

*C. M. Noel.*

### Notes and Queries.

**The Editor is always willing to answer in this column, without any fee, all reasonable questions, as soon as possible.**
**But the following rules must be carefully observed :—**

1. **Every communication must be accompanied by the name and address of the writer.**
2. **The question must always bear upon nursing, directly or indirectly.**

**If an answer is required by letter a fee of half-a-crown must be enclosed with the note containing the inquiry, and we cannot undertake to forward letters addressed to correspondents making inquiries. It is therefore requested that our readers will not enclose either a stamp or a stamped envelope.**

*Indian Nursing Service.*

(50) 1. To whom should I apply for information about the Indian Nursing Service? 2. Is the L.O.S. examination hard, and could I prepare for it by correspondence ?—*R. B. N. A.*

1. Apply to the India Office, St. James's Park, S.W., for form containing Memorandum of Information. 2. You can be prepared for the theoretical part of the L.O.S. examination by correspondence, but you must have practical instruction in the technical portion.

Will you kindly tell me to whom I should apply with regard to the Indian Army Nursing Service ?—*Cambridge.*

See reply to *R. B. N. A.*

*Dietary.*

(51) Can you recommend me a work on dietary for different complaints such as rheumatism, diabetes, etc., which gives a list of articles which patients may, and may not, eat ?—*Private Nurse.*

"The Art of Feeding the Invalid," by a medical practitioner, and a lady professor of cookery might suit you. Mrs. Ernest Hart's "Diet in Sickness and in Health" is also a very useful publication. Both books may be obtained from the Scientific Press.

*Patent.*

(52) I have thought of a new bed-pan suitable for cases of fracture of the legs. Can you tell me of anyone who would be willing to buy the idea, as I cannot afford to get it patented myself ?—*Aidyl.*

Apply to either Messrs. W. H. Bailey and Son, 38 Oxford Street, W., or Messrs. Stonier and Co., 78 Lord Street, Liverpool.

*Custom.*

(53) Will you tell me if it is usual for the district nurse to be excluded from the committee meetings of the association employing her, and also if she may appear at the annual public meeting, or not ?—*A. T. M.*

The district nurse has no right to appear at any of the meetings of the association which employs her, and it is not usual for her to do so.

*Massage.*

(54) Will you kindly tell me if I can obtain a course of lessons and a certificate in massage in Huddersfield ?—*Alma.*

Write to the Secretary, the Incorporated Society of Trained Masseuses, 12 Buckingham Street, Strand, London, W.C.

I would be glad of the name of any institute or teacher in Manchester from whom I could receive a course of instruction and a good certificate in massage.—*I. B.*

Write for the name of a teacher to the Society of Trained Masseuses, 12 Buckingham Street, Strand, London, W.C.

*Library.*

(55) Is there a circulating library of medical books by authors of the present day, from which nurses who are subscribers can get books which are useful to them ? Nurses in hospitals have the advantage of the libraries attached to the hospital, but nurses who are unattached to large institutions have not.—*G. M. S.*

You can obtain books of any kind from such libraries as Mudie's, and medical books from Lewis'. The Trained Nurses' Club, 12 Buckingham Street, Strand, W.C. : and the Royal British Nurses' Association, 10 Orchard Street, W., have libraries for the benefit of their members.

**Standard Books of Reference.**

**"The Nursing Profession : How and Where to Train." 2s. net ; post free 2s. 4d.**
**"Burdett's Official Nursing Directory." 3s. net ; post free, 3s. 4d.**
**"Burdett's Hospitals and Charities." 5s.**
**"The Nurses' Dictionary of Medical Terms." 2s.**
**"Burdett's Series of Nursing Text-Books." 1s. each.**
**"A Handbook for Nurses." (Illustrated). 5s.**
**"Nursing : Its Theory and Practice." New Edition. 3s. 6d.**
**"Helps in Sickness and to Health." Fifteenth Thousand. 5s.**
**"The Physiological Feeding of Infants." 1s.**
**"The Physiological Nursery Chart." 1s. ; post free, 1s. 3d.**
**"Hospital Expenditure : The Commissariat." 2s. 6d.**
**All these are published by the Scientific Press, Ltd., and may be obtained through any bookseller or direct from the publishers, 28 and 29 Southampton Street, London, W.C.**

### Travel Notes.

By Our Travelling Correspondent.

### XCIX.—TEN DAYS IN FRIESLAND AND GRONINGEN.

If, as is probable, you know North and South Holland, you might very profitably spend a week or ten days in Friesland. It is very little known to the general touring world, and though the scenery is flat and little varied, it cannot be called uninteresting because it is so different to anything we are accustomed to, and the means of getting about by canals, etc., is quite unique.

There are two principal ways of entering the province, either by Stavoren or Harlingen, and the former is, I think, the more convenient, especially if you combine this trip with one to North Holland. From Enkhvizen you cross to Stavoren in an hour and a quarter, thus having a little taste of the chopping waves of the Zuyder Zee. At Stavoren itself there is but little to see; it was once a very important port, and so rich was the town that tradition says the locks and hinges of doors were made of pure gold. The decay of the place is attributed by chroniclers to the reckless conduct of a wealthy lady of the town, and Guicciardini gives us the following account of the circumstance: "There was a widow in the said town so rich that she knew not the extent of her wealth, which rendered her petulant and saucy. She freighted a ship for Dantzig, with orders to its master to bring back the most exquisite and rare produce in exchange for the merchandise that she sent; and as the master of the ship found there no merchandise more in requisition than grain, he returned laden with it to Stavoren. This so displeased the widow that she ordered the ship master, if he had loaded the said corn at Backboort, that he should throw it into the sea at Stuerboort, that is to say, if he had received it on the poop, he should throw it over the prow. This being done at the instant, in that very place, there arose so great a sandbank, that the harbour was so blocked, that since no great ships could enter it and its traffic and commerce decayed."

It is quite true that the huge sandbank *does* block the harbour, and that it is called *vrouwensand*, or lady's sand.

### Costume in Hindeloopen.

If you can manage an hour or two in Hindeloopen try to do so to see the costumes. It was there I saw a very strange kind of jacket worn by the women, with immensely high peaks over the shoulders like upstanding epaulettes, and the colours of the clothes generally are much gayer than in North Holland. The Friesland women have a great reputation for beauty; their complexions are delicately fair and the hair generally flaxen. They are rather heavily built, but fine Juno-like figures, stately and dignified, and their skating is perfection.

### Groningen.

I think you would do well to make your headquarters at Groningen and Leeuwarden. The former is the more interesting from its historical associations. In 1580 Groningen fell into the hands of the Spaniards through the treachery of Renneberg, governor of Friesland, a trusted friend of the Prince of Orange. Renneberg stipulated to deliver the town to the Spanish King on receipt of 20,000 crowns and a yearly pension of 10,000 florins; moreover, the King was to use all his influence to bring about the marriage of Renneberg with a widow, Countess Berlaymont, who had long been the object of this love-sick traitor's matrimonial design. One feels glad to know that this high-born ruffian, though he achieved the money, did not marry the lady. The city was formally delivered up to his Majesty's Government, but the Prince of Orange at once commenced a siege, under his brother Maurice, which continued a short time, but was eventually raised in consequence of the defeat of the Stadholder's troops at Hardenburg Heath. Eventually it was again invested, and surrendered to the Nassau general after a revolt of some two months.

### Churches in Groningen.

In Protestant countries these are usually wanting in interest; St. Martin is fine, built early in the seventeenth century, and so is the Aakerk. The Roman Catholics worship in the Prœderkerk, and the Mass there is very grand, the voices and music reverent and subdued instead of the nasal bawl which so often spoils their beautiful office. The Groote Markt is immense, the biggest in the Netherlands, I believe. Near to St. Martin's is an extremely beautiful house of the fourteenth century, which used to be a kind of court-house; there are several good specimens of domestic architecture in Groningen, chiefly in the Burgstraal. Present-day guide-books say little of Groningen and its strange old buildings, but in reality there is much to be seen there, and artists and photographers would delight in it. I was led there first by reading M. Havard's books, "The Dead Cities of the Zuyder Zee," "Picturesque Holland," etc.

### Leeuwarden and Harlingen.

The architecture here is not equal to that of Groningen. The town hall only dates from 1715, and there are two ancient towers, one of them very much out of the perpendicular, but the gem of Leeuwarden is the "chancellerie.' M. Havard regretted much being refused permission to explore the interior, and as the forbidden always presents attractions, I tried hard to soften the hard heart of the guardian, but entirely without success. It remains with me a lost opportunity and always regretted. Harlingen can be visited from Leeuwarden and is not worth a residence. The approach from the Zuyder Zee is picturesque; there are two towers, some primitive windmills, and a curious rather than handsome water-gate. It is, however, a very busy part, and there is much trade with England carried on there.

Another place to visit from Leeuwarden is Dokkum; it is a large flax-selling centre, and there is an immense quantity of butter and cheese made there, the pastures so used being extremely rich, but the peasants are too conservative to avail themselves of modern machinery, with the result that their farms are not so productive as those of many other parts of Friesland, in spite of the extreme richness of the soil.

### Meppel, Kampen, Zwolle.

In returning home you might, by taking a circular ticket, come through the above towns. Meppel is not specially interesting, and perhaps you will choose to stop at Assen instead, but Zwolle, with Kampen to be visited from it in a day, is worth seeing. There is a most beautiful gateway there called the Sassenpoort, square, with four pointed turrets at the corners and a different shaped one in the middle. There are two fine churches, Notre Dame and St. Michel.

Kampen is on the whole more picturesque, and presents better corners to the artist. There, too, is a strange gate, almost Japanese looking, called the Cellebroederspoort.

Another place easy of access from here is Deventer, a very short day's excursion from Zwolle, and full of interest.

### TRAVEL NOTES AND QUERIES.

Schwalbach for Anæmia (Gladys).—It is a small place and much quieter than many of the Continental spas. The annual number of visitors is about 6,000, and there is a good kurhaus, where there are dances, concerts, and fêtes of all kinds. The band, which plays twice a day in the gardens near to the springs, is good. It is advisable to go in June, if possible; by the end of July the baths, and springs are much crowded, and it is difficult to secure a time at the bath-house except at unseasonable hours No, the doctors' charges are not at all high. The walks are very beautiful round, but as a rule those undergoing the cure are not allowed to make much exertion.

